# Enhancing tunnel crack detection with linear seam using mixed stride convolution and attention mechanism

**DOI:** 10.1038/s41598-024-65909-1

**Published:** 2024-07-01

**Authors:** Lang Lang, Xiao-qin Chen, Qiang Zhou

**Affiliations:** 1 School of Intelligent Manufacturing, Chongqing Three Gorges Vocational College, Chongqing, 404155 China; 2 School of Computer Science and Technology, Chongqing University of Posts and Telecommunications, Chongqing, 400065 China

**Keywords:** Attention mechanism, Mixed strip convolution, Crack detection, Linear seams, Civil engineering, Electrical and electronic engineering

## Abstract

Cracks in tunnel lining structures constitute a common and serious problem that jeopardizes the safety of traffic and the durability of the tunnel. The similarity between lining seams and cracks in terms of strength and morphological characteristics renders the detection of cracks in tunnel lining structures challenging. To address this issue, a new deep learning-based method for crack detection in tunnel lining structures is proposed. First, an improved attention mechanism is introduced for the morphological features of lining seams, which not only aggregates global spatial information but also features along two dimensions, height and width, to mine more long-distance feature information. Furthermore, a mixed strip convolution module leveraging four different directions of strip convolution is proposed. This module captures remote contextual information from various angles to avoid interference from background pixels. To evaluate the proposed approach, the two modules are integrated into a U-shaped network, and experiments are conducted on Tunnel200, a tunnel lining crack dataset, as well as the publicly available crack datasets Crack500 and DeepCrack. The results show that the approach outperforms existing methods and achieves superior performance on these datasets.

## Introduction

The advancement of tunnels has significantly improved traffic efficiency and contributed to economic growth. However, initial tunnels were susceptible to cracks and other structural defects that, if left unaddressed, posed substantial risks to travel safety. To mitigate these risks, the development of efficient and automated crack detection technologies has tremendous potential for application^1^.

Historically, crack detection has primarily relied on digital image processing techniques such as wavelet transform, percolation models, and minimum path methods. These methods leverage the unique morphology and strength characteristics of cracks. Subirats et al. employed wavelet transform to successfully distinguish cracks from noise by analyzing frequency subbands^2^. Similarly, Qu et al. utilized a percolation model to enhance the identification and continuity of fine cracks while minimizing noise^3^. Research has also incorporated techniques like the free anisotropic shortest path method, which detects cracks of any shape and orientation by considering both strength and shape features^4^, and Kaul et al.’s approach using a minimum path-based algorithm for complex curve topologies^5^. While traditional crack detection methods such as wavelet transform, percolation models, and minimum path techniques prove effective in scenarios with simple backgrounds and single-structure cracks, they often falter in more complex environments. These methods primarily rely on local information for feature extraction, thereby constraining their capacity to discern global features and limiting their effectiveness in complex backgrounds. In response to these limitations, recent advancements have prompted a shift towards machine learning techniques, including random forest^6^, SVM, and AdaBoost. These methods utilize manually designed feature extractors to improve crack detection accuracy. However, they still face challenges in differentiating between crack features and background disturbances in intricate settings, often resulting in false detections and increased noise^7^. In tunnel lining structures, Qu et al. introduced a sophisticated multi-step approach focused on detecting, marking, and removing lining seams^1,8^.However, these methods primarily rely on local feature information, resulting in limitations in capturing global information. As a result, the resulting images of cracks are often plagued by noise and incompleteness.

Rapid advancements in deep learning for automatic feature extraction have profoundly influenced crack detection research. Current deep learning methods primarily utilize networks such as HED^9^ and U-Net^10^, which, despite their effectiveness, often struggle to differentiate between lining joints and cracks in tunnel structures due to their similar appearances. To address these challenges, several enhancements have been made to these networks. For instance, Liu et al.^11^ enhanced the HED network by implementing a deeply supervised training strategy and integrating Guided Filters and Conditional Random Fields to refine detection details. Yang et al.^12^ incorporated a feature pyramid and hierarchical boosting module to enhance detection accuracy. Similarly, enhancements to the U-Net network include Han et al.’s^13^ implementation of a round-trip sampling block in place of the traditional skip-layer connections, and Zhou et al.^14^ who introduced a mixed attention module and a multi-scale feature fusion strategy within the skip-layer connections to enhance performance in detecting cracks in tunnel linings. These developments represent a concerted and strategic effort to overcome the limitations of current deep learning approaches in distinguishing structural elements in complex environments.

However, in environments with lining structures, these methods often fail to effectively differentiate between lining seams and cracks, leading to frequent cases of false detections. To address this, many researchers have proposed using attention mechanisms and strip convolutions to improve performance, specifically targeting objects with unique shapes. Attention mechanisms are typically divided into three types: channel, spatial, and self-attention. Channel attention, as utilized in SENet^15^ and ECANet^16^, prioritizes relevant image channels, while spatial attention mechanisms, such as those in Non-Local neural networks^17^, focus on specific image regions. Additionally, models such as the Convolutional bottleneck attention module (CBAM)^18^, Dual Attention^19^, Multidimensional Collaborative Attention Module^20^ leverage these approaches, using sequential or parallel execution strategies to optimize feature integration. Strip convolution enables models to focus more on features in a specific direction of an image (such as horizontal or vertical), thus effectively complementing global features. Many models have implemented this method, including the Inception architecture^21^, which suggests decomposing an $N\times N$ convolutional layer into two layers of $1\times N$ and $N\times 1$ to improve computational efficiency. Additionally, strip convolution has been utilized across various specific fields. Mei et al^22^. introduced a strip convolution module for road segmentation scenarios to capture long-distance dependencies and enhance segmentation performance. Zhou et al^23^ . employed strip convolution to collect more detailed features of cracks, acting as a robust complement to features extracted by conventional convolutions. Yang et al^24^.developed a multi-scale feature convolution attention network, named MSFCA-Net, utilizing different sizes of strip convolutions to segment field crops and weeds. Liao and colleagues^25^ harnessed strip convolution to improve feature extraction of rice seedling leaves, thereby providing strong support for the development of intelligent weeding technologies.

**Figure 1:**
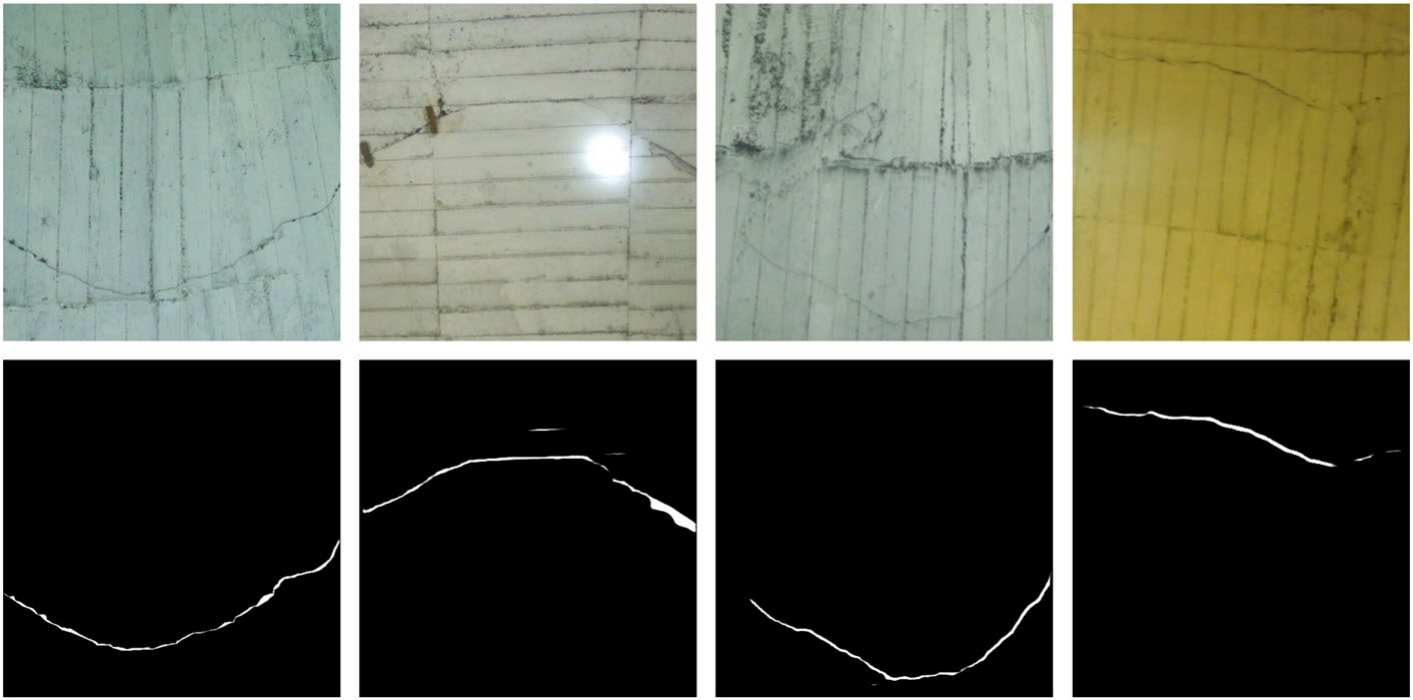
An illustrative diagram depicting crack detection in a tunnel lining structure. The top row displays the original image, while the bottom row showcases the detection results achieved by the method proposed in this paper.

To address the limitations of existing crack detection methods, a new deep learning-based tunnel lining crack detection method based on attention mechanisms and strip convolution. As shown in Fig. 1, the morphological features of cracks and lining seams are very similar, making it easy to mistakenly detect lining seams as cracks. It was observed that lining seams mostly appear in horizontal or vertical shapes, while cracks often appear as curves. Therefore, starting from the shape characteristics of lining seams and cracks, an improved attention module and mixed strip convolution. The improved attention module adopts a multi-branch structure, especially aggregating features in the height (H) and width (W) dimensions, which can effectively capture long-distance features in horizontal and vertical directions to distinguish between cracks and lining seams. On the other hand, based on the morphological characteristics of cracks, to enhance feature extraction, mixed strip convolution has been implemented. This method builds upon the foundational horizontal and vertical strip convolutions by incorporating diagonal strip convolutions in both left and right directions. Such an approach enables the further capture of long-distance dependencies of cracks, facilitating the differentiation between cracks and lining seams.

**Figure 2:**
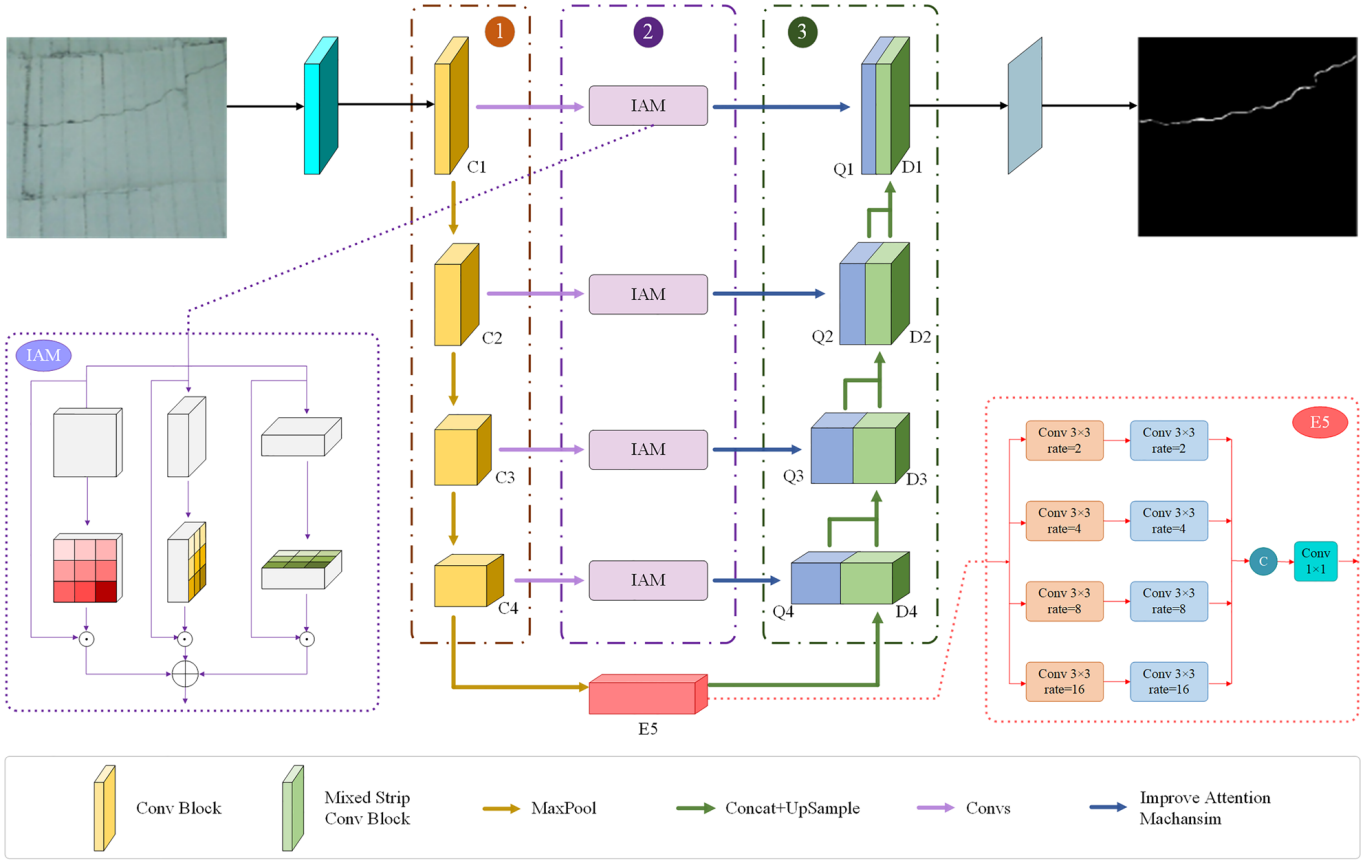
The comprehensive structure of the proposed network is depicted. The network can be dissected into three principal components: the encoding phase, the skip connection segment, and the decoding phase. Within the skip connection segment, we have incorporated an enhanced attention mechanism to bolster the network’s feature extraction capabilities. The receptive field enhancement module, employing parallel dilated convolutions, is applied to the lower and red blocks of the network to augment the network’s receptive field. In the decoding stage, a mixed strip convolution module is implemented to capture the characteristics of both crack and lining joints better. Finally, the network’s output is $D_{1}$.

Our method incorporates several key modules to enhance the network’s performance in recognizing cracks and lining seams. The overall network structure is depicted in Fig. 2. Firstly, an improved attention module is introduced that captures long-range dependencies between cracks and lining seams. Drawing on the attention placement in networks from the DA-TransUNet^26^ and AttentionU-Net^27^ methods, this module is seamlessly integrated into the network, positioned between the encoder and decoder of the U-shaped network, to enhance the network’s ability to recognize lining seam features. Furthermore, to effectively address the challenges posed by the large, narrow, and continuous distribution of cracks, a mixed strip convolution module is incorporated in the decoder. This module employs four strip convolutions, including horizontal, vertical, left diagonal, and right diagonal directions, to capture remote contextual information and minimize interference from irrelevant regions. By integrating these proposed modules into the U-shape structure, our method can accurately detect the cracked areas in tunnel lining structures and improve safety during tunnel operation.

The contributions to the field of tunnel lining crack detection include the following: (1) Based on the morphological characteristics of lining seams, an improved attention mechanism is proposed that effectively distinguishes between crack and lining seam features by aggregating features along two spatial dimensions (height and width) and complementing global spatial feature information. (2) Based on the morphological characteristics of cracks, a mixed strip convolution module is employed that captures remote contextual information in four distinct directions and mitigates interference from irrelevant regions. (3) A novel deep learning-based crack detection network is introduced that surpasses existing methods in performance on the Tunnel200 dataset and demonstrates strong performance on the publicly available DeepCrack and Crack500 datasets.

The structure of the remaining sections in this paper is as follows: “Introduction” presents a summary of related work on crack detection and attention mechanisms. “The proposed methods” provides a detailed description of the proposed network. “Experimental results and analysis” contains details about the dataset, evaluation metrics, implementation specifics, and a series of experiments aimed at evaluating the performance of the crack detection method in tunnel lining structures. Lastly, “Conclusion” concludes the paper and offers insights into future research directions.

## The proposed methods

### Improved attention mechanism

**Figure 3:**
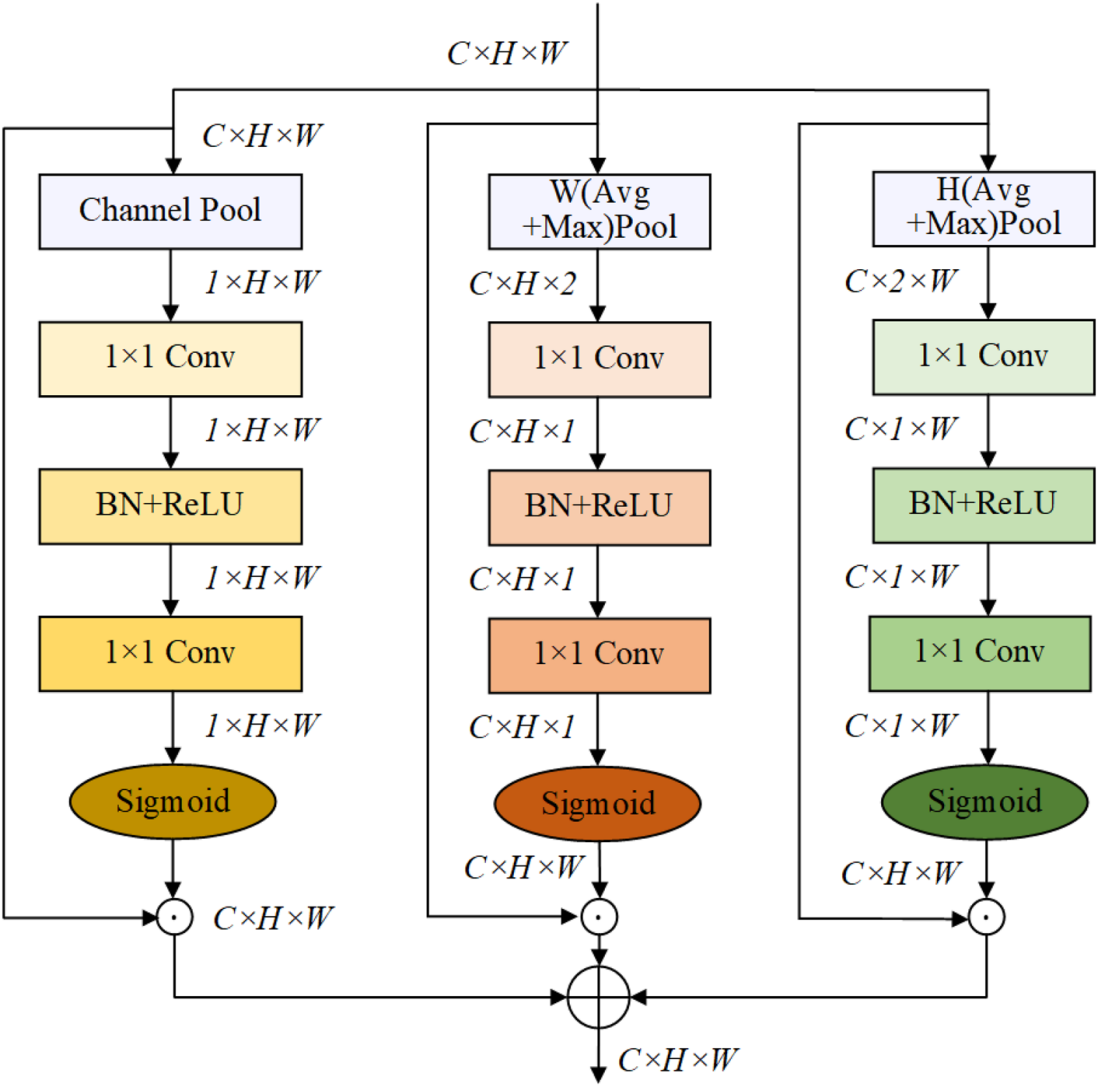
The structural diagram of the proposed improved attention mechanism module.

In this section, an improved attention mechanism is proposed that captures both channel attention and spatial attention through a three-branch structure, as illustrated in Fig. 3. The initial branch utilizes channel pooling to efficiently distribute weights across distinct spatial locations, while the remaining two branches focus on the interplay between channels and the height (H) and width (W) dimensions of the input tensor, respectively. In conventional approaches, the potential interactions between channel attention and spatial attention often go unexplored. To rectify this limitation, an innovative concept of cross-dimension interaction is introduced, drawing inspiration from the methodology employed in constructing spatial attention mechanisms. This novel approach effectively captures the interplay between the spatial dimensions and the channel dimension of the input tensor, culminating in a more cohesive and comprehensive model.

The proportion of pixels that display cracks in the entire image is relatively low, and a significant portion of these pixels exhibit elongated structures. As a result, directly applying the existing spatial attention mechanism proves to have limited efficacy in the task of crack detection. To tackle this challenge, an attention mechanism for feature aggregation has been devised those functions in both the horizontal and vertical directions. This approach enables the acquisition of more precise spatial feature information and the accommodation of the long-range dependencies inherent in cracks. The feature aggregation process involves pooling the input feature X using (H,1) or (1,W) pooling kernels sized to the image dimensions. The aggregated features along the height direction are expressed as follows:

1 $$\begin{aligned} H_{Pool}=[MaxPool(X_H),AvgPool(X_H)], \end{aligned}$$

2 $$\begin{aligned} W_{Pool}=[MaxPool(X_W),AvgPool(X_W)], \end{aligned}$$

where $X_H$ and $X_W$ represent the feature map obtained by pooling the input feature along the height and width dimensions, respectively. Then, a $1 \times 1$ convolutional layer is applied to transform the features, resulting in:

3 $$\begin{aligned} g^{H}=\sigma \left( C_{2}(\delta \left( C_{1}\left( HPool\right) \right) \right) , \end{aligned}$$

4 $$\begin{aligned} g^{W}=\sigma \left( C_{2}(\delta \left( C_{1}\left( WPool\right) \right) \right) , \end{aligned}$$

wherein $C_{1}$ and $C_{2}$ denote the $1\times 1$ convolutional transformations, $\delta$ signifies the ReLU activation function, and $\sigma$ denotes the sigmoid function. The output $Y_{1}$ can be represented as follows:

5 $$\begin{aligned} Y_{1} = X g^{H} \end{aligned}$$

6 $$\begin{aligned} Y_{2} = X g^{W}. \end{aligned}$$

The first step in the overall spatial attention involves channel pooling, followed by convolution, batch normalization, and ReLU and sigmoid activations to obtain the corresponding weights:

7 $$\begin{aligned} \begin{aligned} S=F_{\text{s}}(X, \theta )&=\sigma \left( W_{2} \delta \left( W_{1} {\text {ChanelPool}}(X)\right) \right) , \end{aligned} \end{aligned}$$

8 $$\begin{aligned} Y_{s} =S X. \end{aligned}$$

The final output *Y* is defined as follows:

9 $$\begin{aligned} Y = Y_{1}+ Y_{2}+ Y_{s}. \end{aligned}$$

### Mixed strip convolution

**Figure 4:**
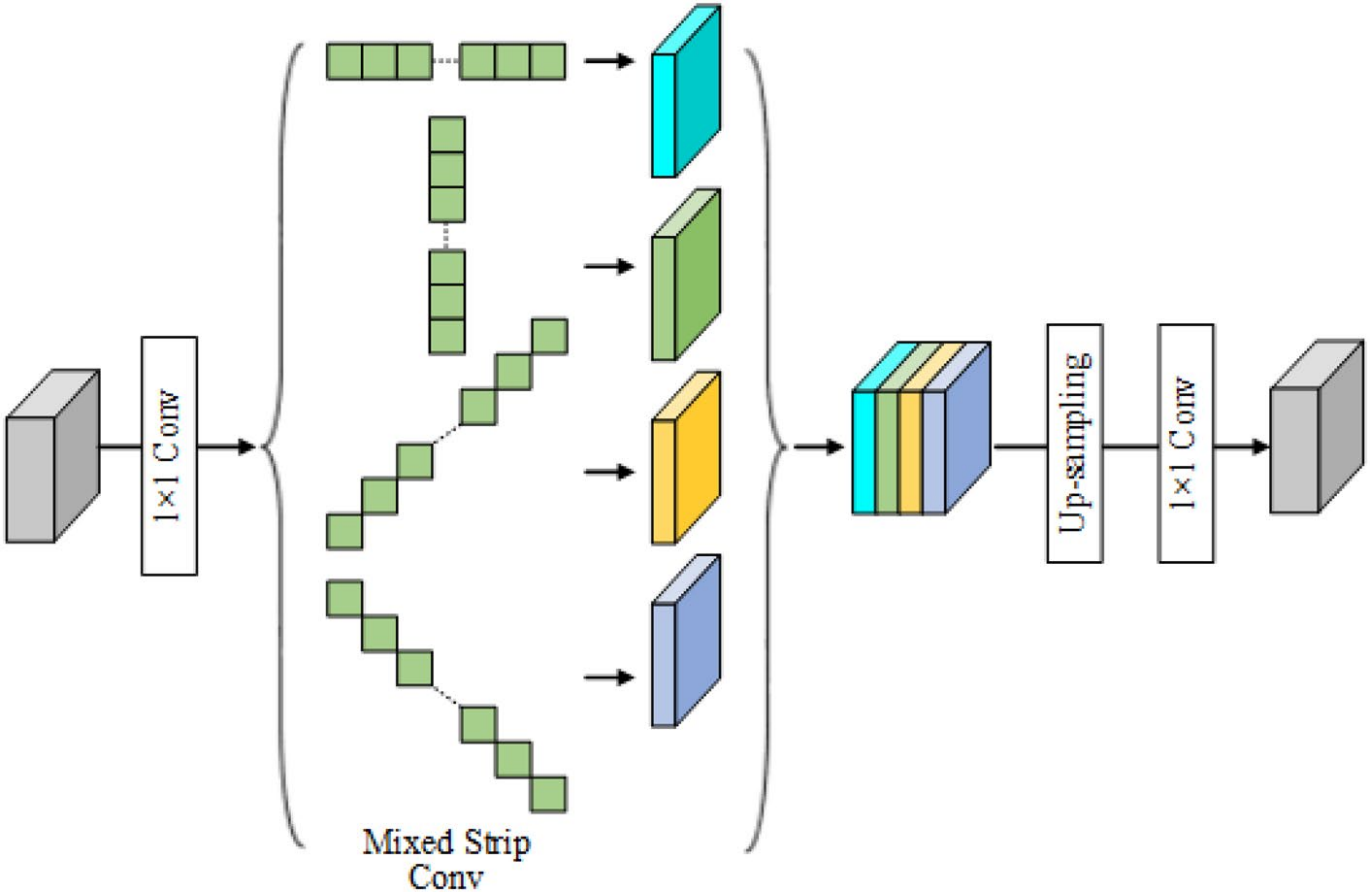
The structure diagram of the proposed mixed strip convolution module.

Conventional CNN networks use square convolutional kernels to learn feature maps, which are suitable for most natural images with blocky shapes. However, crack images are characterized by their large span, long stripes, and continuous distribution. Square convolution fails to adequately capture the linear characteristics of cracks, resulting in the inclusion of extraneous information from adjacent pixels. Strip convolution, which uses long stripes along one spatial direction to capture the long dependencies in the crack region, is more consistent with the morphological characteristics of cracks and lining seams^28^. Furthermore, it captures local context along another spatial direction and mitigates the influence of irrelevant regions on feature learning. To tackle these challenges, the Mixed Strip Convolution Module (MSCM) has been incorporated into the crack detection process, building upon prior research. As depicted in Fig. 4, MSCM captures long-range dependencies in crack information from four distinct directions through horizontal, vertical, left diagonal, and right diagonal strip convolutions.

The feature map is represented by the input $X \in \mathbb {R}^{H \times W \times C}$, where *H*, *W*, and *C* stand for the height, width, and number of channels, respectively. To prepare the feature map for processing, a $1 \times 1$ convolutional layer is applied to adjust its dimensions. Subsequently, the adjusted feature map is fed into four parallel processing branches, each specializing in feature learning along a different direction. Finally, the different feature maps obtained from the four branches are stitched together, upsampled, and convolved by a $1 \times 1$ layer to generate the final feature map output. Let $w \in \mathbb {R}^{2k+1}$ be a strip convolution filter of size $2k + 1$, $D=(D_h,D_w)$ be the direction of the filter *w*, and $Z_D \in \mathbb {R}^{H \times W \times C'}$ denote the result of strip convolution. The strip convolution can be defined as follows:

10 $$\begin{aligned} Z_D[i,j]=(X*W)D[i,j]={\sum \limits _{l = -k}^k} x[i+D_h^l,j+D_wl] \cdot w[k-l], \end{aligned}$$

where $X *w$ denotes the convolution operation. The direction vector of the strip convolution is represented by *D*, where the values (0,1), (1,0), (1,1), and (-1,1) correspond to the horizontal, vertical, left diagonal, and right diagonal convolutions, respectively. To ensure consistency with the $3 \times 3$ convolution kernel, k is set to 4 for the filter w, resulting in each strip convolution having nine parameters.

### Receptive filed enhance module

The size of the receptive field has a significant impact on a network’s ability to perceive target size, with smaller receptive fields being better suited to recognizing smaller targets and larger receptive fields being more adept at recognizing larger targets. The RFE module, which is based on ASPP^29^, features four branches that use the same hole rate of^2,4,8,16^. Each branch utilizes $3 \times 3$ convolutions with the same dilation rate to achieve an expanded receptive field. To align with the network’s channel dimension, the number of channels in the second convolution of each branch is configured to be 128. Ultimately, the outcomes from the different branches are amalgamated, and the ultimate multi-scale feature map is generated using a $1 \times 1$ convolution.

### Loss function

The conventional binary cross-entropy (BCE) loss function is widely used in crack detection. Nonetheless, its application presents challenges, as the number of crack pixels is often significantly lower than that of the background pixels. Using the standard BCE loss during training may cause the model to predominantly emphasize the non-crack pixels, due to their prevalence in the dataset. Consequently, the model may inadvertently acquire features primarily associated with class samples containing a large number of pixels, potentially degrading its performance in crack detection. To address this issue, a weighted BCE loss function is employed. The loss function is defined as follows:

11 $$\begin{aligned} \begin{aligned} L_{wbce}\left( W \right) =&-w_{0} \sum _{j \in Y_{+}} \log {\text {Pr}}\left( y_{j}=1 \mid X, W\right) \\&-w_{1} \sum _{j \in Y_{-}} \log {\text {Pr}}\left( y_{j}=0 \mid X, W \right) , \end{aligned} \end{aligned}$$

where ${Y_ + }$ and ${Y_ - }$ represent the samples containing cracks and those without cracks, respectively. Furthermore, ${w_0}$ and ${w_1}$ are used to denote the weights assigned to crack and non-crack pixels, respectively. ${w_0}$ is defined as ${w_0} = \frac{{\left| {{Y_ - }} \right| }}{{\left| {{Y_ + }} \right| }}$ and ${w_1}$ as ${w_1} = 1$, with $\left| {{Y_ + }} \right|$ and $\left| {{Y_ - }} \right|$ representing the total count of crack and non-crack pixels in the entire training dataset.

## Experimental results and analysis

Both the proposed method and the compared method were implemented using the PyTorch framework. In the training phase, the images in the dataset were standardized to a size of 448 $\times$ 448 pixels. The batch size was set to 4, and the learning rate was set to 1e−4. The Tunnel200 dataset underwent training for 100 epochs, while both the Crack500 and DeepCrack datasets were trained for 300 epochs each. During the decoder stage, upsampling was carried out using the bilinear interpolation method. Batch normalization and ReLU activation were applied in each convolutional layer during both the encoder and decoder stages. The optimizer used was adaptive moment estimation (Adam) with a weight decay of 1e−3. The experiments were conducted on an Ubuntu 16.04 system equipped with a 4-core Intel(R) Xeon(R) Silver CPU and a Tesla V100 32GB GPU.

### Datasets

In this paper, the efficacy of the proposed model in detecting cracks in tunnel lining structures is demonstrated, substantiated by experimental results obtained from the Tunnel200 dataset. Furthermore, to exemplify the effectiveness of our proposed approach, it is validated using the publicly accessible DeepCrack and Crack500 crack datasets. Here is a concise overview of these three datasets.

1. *Tunnel200*^14^ : this dataset was captured by Zhou et al. using a cell phone on a real tunnel lining surface. The dataset presents significant difficulties in detecting cracks due to severe interference from tunnel light, illumination, and lining seams. T The original image size in the dataset is $2048 \times 1536$, and to reduce computational effort, the authors uniformly cropped the images to $448 \times 448$. The data consists of only 200 images, of which we use 180 as the training set and 20 as the test set. Considering the limited size of this dataset, the 10-fold validation methodology outlined in the original paper is adopted, and the final result is derived by averaging the outcomes from these 10 folds.
2. *CRACK500*^12^: the CRACK500 dataset was collected by the Temple University team, who employed cell phones to annotate crack defects in intricate pavements. The resolution of the original images is $2000 \times 1500$, and a cropping method is used to divide the image into 16 non-overlapping regions, generating 3368 images of $448 \times 448$ pixels. In this paper, 3000 of these images are utilized for training and 368 are reserved for testing.
3. *DeepCrack*^11^: this dataset is a well-established publicly available pavement crack detection dataset, extensively employed for validating the efficacy of algorithms in the realm of crack detection. In this study, the DeepCrack dataset is augmented by integrating the CFD dataset, resulting in a combined dataset comprising 758 images. The dataset is partitioned into 521 images for the training set and 237 for the testing set.

### Comparison methods

This paper conducted comparative experiments with a wide array of methods. The following section will provide a brief introduction to the methods that were compared.

1. *HED*^9^: this method is based on the full convolutional neural network FCN and incorporates a deep supervision strategy to enhance model performance. Many current crack detection models are built upon this network.
2. *U-Net*^10^: this method, commonly used in medical image segmentation, utilizes a U-shaped encoder-decoder architecture and skip connections for feature fusion. Many current crack detection models are based on this network.
3. *DeepLabV3+*^30^: this method is a classic network for semantic segmentation and proposes the concept of null convolution to improve the perceptual field and increase global information acquisition without increasing model parameters.
4. *DeepCrack*^11^: this method, which builds upon the HED network, incorporates guided filters and conditional random fields to enhance the final detection performance.
5. *DeepCrackT*^31^: an improvement on the U-Net network, this method fuses encoder and decoder features to improve crack detection results.
6. *FPHBN*^12^: This methodincorporates feature pyramid and hierarchy boosting modules into the HED network, aiming to enhance feature propagation and improve network learning.
7. *HACNet*^32^: this method incorporates feature pyramid and hierarchy boosting modules into the HED network, aiming to enhance feature propagation and improve network learning.
8. *SwinTransformer*^33^: this approach has gained popularity in the field of visual Transformers and has demonstrated promising outcomes in segmentation and detection tasks. In this study, the abbreviation “SwinT” is employed to refer to this method.
9. *TransUnet*^34^: this method incorporates a Transformer structure into the U-shape network, enhancing the model’s capacity for contextual modeling. It has demonstrated promising results in the domain of medical image segmentation.
10. *ECDFFNet*^23^: this method is currently the method with better results in the field of crack detection. It proposes Enhanced Convolution and Dynamic Feature Fusion strategies to improve the final detection performance.
11. *TCDNet*^14^ this approach stands as the leading method in the field of crack detection, boasting superior performance. It introduces Enhanced Convolution and Dynamic Feature Fusion strategies aimed at enhancing the final detection performance.

### Evaluation metrics

In this study, $precision$ (*P*), $recall$ (*R*), the *P*-*R* curve, and the *F1-score* ($F_{1}$) are employed as evaluation metrics for assessing the performance of these models. $Precision$ (*P*), $recall$ (*R*), and *F1-score* ($F_{1}$) are commonly used evaluation metrics for classification models. Precision quantifies the ratio of true positive predictions to all positive predictions made by the model, while recall quantifies the ratio of true positive predictions to all actual positive instances in the dataset. The F1-score represents the harmonic mean of precision and recall, offering a comprehensive performance metric that considers both aspects.

12 $$\begin{aligned} {Precision (P)} = \frac{{TP}}{{TP + FP}}, \end{aligned}$$

13 $$\begin{aligned} {Recall (R)} = \frac{{TP}}{{TP + FN}}, \end{aligned}$$

14 $$\begin{aligned} {F_{1} } = \frac{{2 \times P \times R}}{{P + R}}, \end{aligned}$$

where *TP* represents true positives, *FP* denotes false positives, and *FN* stands for false negatives. Furthermore, in this paper, crack detection is treated as a binary semantic segmentation task, aiming to differentiate the crack region from the background. To assess the models’ performance in this task, three semantic segmentation metrics are utilized: pixel accuracy (*PA*), mean pixel accuracy (*MPA*), and mean intersection over union (*MIoU*).

15 $$\begin{aligned} {PA} = \frac{{\sum \limits _{i = 0}^k} P_{ii}}{{\sum \limits _{i = 0}^k}\sum \limits _{j = 0}^k P_{ii}}, \end{aligned}$$

16 $$\begin{aligned} {MPA} =\frac{1}{{K+1}} \sum \limits _{i = 0}^k \frac{{P_{ii}}}{\sum \limits _{j = 0}^k P_{ij}}, \end{aligned}$$

17 $$\begin{aligned} {MIoU} =\frac{1}{{K+1}} \sum \limits _{i = 0}^k \frac{{P_{ii}}}{\sum \limits _{i = 0}^k P_{ij}+\sum \limits _{i = 0}^k P_{ji}-P_{ii}}, \end{aligned}$$

where *K* represents the number of classes (in this case, $K=2$ for crack and non-crack), and $p_{ij}$ signifies the count of pixels of class *i* predicted to belong to class *j*. Additionally, beyond the previously mentioned metrics, the processing speed of the models is quantified using the *FPS* (frames per second) metric.

### Experimental results

**Figure 5:**
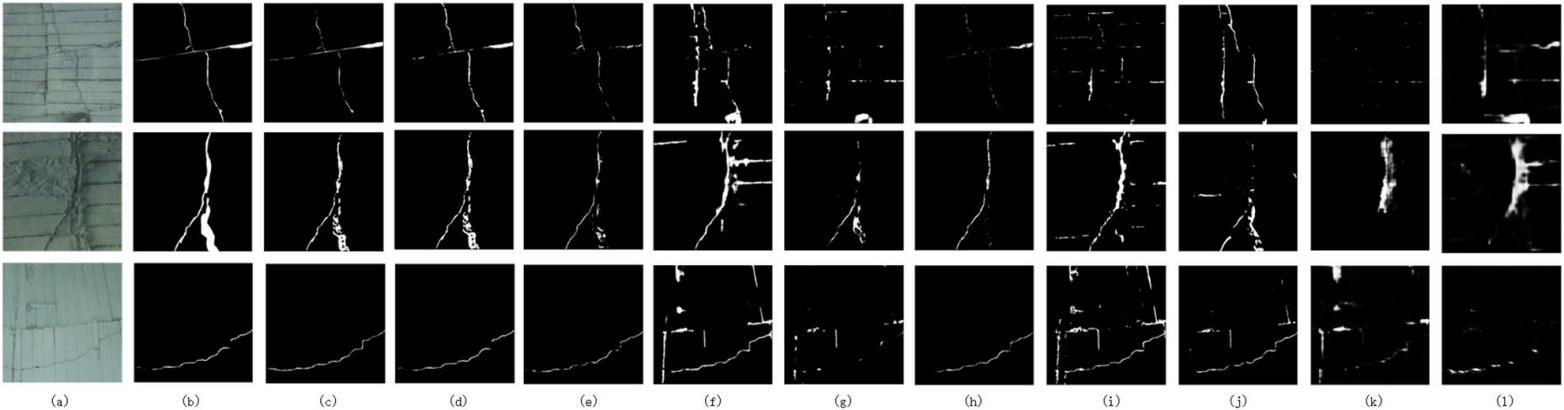
Visualization results obtained by various methods selected from the Tunnel200 dataset. From left to right column: (**a**) raw image, (**b**) ground truth, (**c**) ours, (**d**) TCDNet^13^, (**e**) ECDFFNet^15^, (**f**) FPHBN^12^, (**g**) HACNet^32^, (**h**) DeepCrack^11^, (**i**) DeepCrackT^31^, (**j**) HED^35^, (**k**) SwinT^33^, (**l**) TransU^34^.

**Figure 6:**
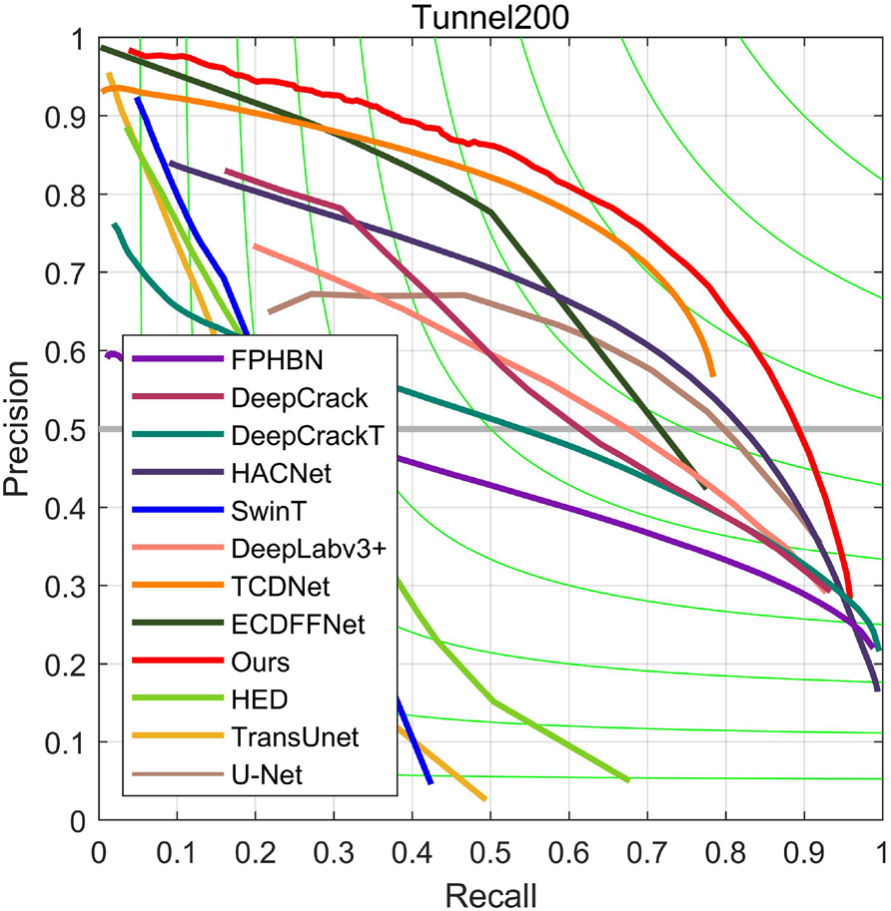
Precision–recall curves on the Tunnel200 test dataset.

1. *Results on Tunnel200* As illustrated in Table 1, our proposed method demonstrates superior performance compared to current crack detection methods on the Tunnel200 dataset, achieving $F_{1}$ and *MIoU* scores of 0.729 and 0.792, respectively.Notably, the TCDNet network, tailored specifically for tunnel crack detection, outperforms other compared methods, attaining $F_{1}$ and *MIoU* values of 0.704 and 0.763, respectively. HACNet gets the highest accuracy. HACNet maintains the same spatial resolution throughout the network architecture, which is particularly important for detecting targets like cracks that have elongated and subtle features. This design minimizes the potential loss of important spatial details during downsampling, while introducing hybrid atrous convolutions to maintain a larger receptive field, thereby enhancing the precision of crack detection. However, this approach does not consider the features of seams, which can lead to misidentifying seam cuts as cracks. In contrast, classical crack detection networks like DeepCrack, DeepCrackT, and FPHBN exhibit comparatively lower performance on this dataset, with $F_{1}$ values of 0.552, 0.536, and 0.451, respectively. Interestingly, the classical U-Net surpasses contemporary crack detection networks on this dataset, achieving an $F_{1}$ score of 0.648. The performance of recent Transformer-based network structures was also assessed, including SwinT and TransUnet, which yielded less satisfactory results due to the dataset’s limited size, with $F_{1}$ scores of only 0.259 and 0.220, respectively. In the Tunnel200 dataset, the proportion of cracks is smaller. This means there are fewer absolute numbers of cracks, presenting a greater challenge for the model to improve recall. The model might miss smaller or less obvious cracks, resulting in a lower recall rate. Visual comparisons of detection results for different models are presented in Fig. 5. Conventional crack detection methods struggle to effectively differentiate between lining seams and crack features, resulting in false positives and missed detections. In contrast, our proposed method demonstrates enhanced accuracy in detecting cracked areas. The Precision–Recall curve in Fig. 6 further illustrates that our proposed method resides in the upper right corner, signifying superior performance compared to other approaches.

**Table 1:** The evaluation metrics of competing methods on the Tunnel200 dataset.

Methods	Tunnel200					
	*P*	*R*	$F_{1}$	*MPA*	*MIoU*	*FPS*
HED^9^	0.408	0.320	0.359	0.652	0.599	**49**
DeepLabV3+^30^	0.655	0.512	0.575	0.721	0.697	18
U-Net^10^	0.723	0.587	0.648	0.756	0.721	35
FPHBN^12^	0.731	0.326	0.451	0.673	0.652	27
HACNet^32^	**0.846**	0.527	0.650	0.765	0.735	25
DeepCrack^11^	0.603	0.508	0.552	0.715	0.689	40
DeepCrackT^31^	0.726	0.425	0.536	0.701	0.668	23
SwinT^33^	0.693	0.159	0.259	0.579	0.566	37
TransUnet^34^	0.511	0.141	0.220	0.569	0.553	11
ECDFFNet^23^	0.783	0.491	0.603	0.749	0.713	17
TCDNet^14^	0.714	0.695	0.704	0.820	0.763	20
**Ours**	0.753	** 0.707**	** 0.729**	** 0.837**	** 0.792**	19

Significant values are in bold.

(2) *Ablation studies* To validate the effectiveness of the proposed modules, ablation experiments were conducted to compare the performance of different modules. Specifically, the U-Net network was used as the Baseline (B) and individually embedded the proposed improved attention mechanism (IAM), mixed strip convolution (MSC), and receptive field enhancement module (RFE) to assess their contributions. As shown in Table 2, the Baseline model had the lowest $F_{1}$ and $MIoU$ scores of 0.648 and 0.721, respectively. However, the addition of the proposed modules effectively improved the Baseline network’s performance, with MSC demonstrating the most significant improvement. Specifically, the $F_{1}$ and $MIoU$ scores improved to 0.684 and 0.754, respectively, indicating that the MSC module can effectively distinguish between crack and lining seam features. Additionally, the proposed IAM module also exhibited significant enhancements in the detection accuracy of the Baseline network.

To further explore the combined effect of the proposed modules, experiments were conducted with different two-by-two combinations and it was found that they can mutually enhance each other’s performance. Specifically, the RFE+MSC approach showed the most significant improvement, with $F_{1}$ and $MIoU$ scores of 0.720 and 0.785, respectively. Finally, by embedding all three proposed modules into the Baseline network, the best experimental results were achieved, with $F_{1}$ and $MIoU$ scores improving to 0.729 and 0.792, respectively.

**Table 2:** Ablation analyze for the proposed architecture on Tunnel200 datasets.

Methods	Tunnel200					
	*P*	*R*	$F_{1}$	*MPA*	*MIoU*	*FPS*
BaseLine (B)^10^	0.723	0.587	0.648	0.756	0.721	**35**
B+RFE	0.731	0.627	0.675	0.782	0.734	29
B+IAM	0.731	0.623	0.672	0.779	0.737	26
B+MSC	0.727	0.645	0.684	0.802	0.754	24
B+MSC+IAM	**0.754**	0.663	0.706	0.819	0.781	24
B+RFE+MSC	0.749	0.692	0.720	0.831	0.785	22
B+IAM+RFE	0.728	0.673	0.699	0.812	0.749	25
**Ours**	0.753	**0.707**	**0.729**	**0.837**	**0.792**	19

Significant values are in bold.

**Figure 7:**
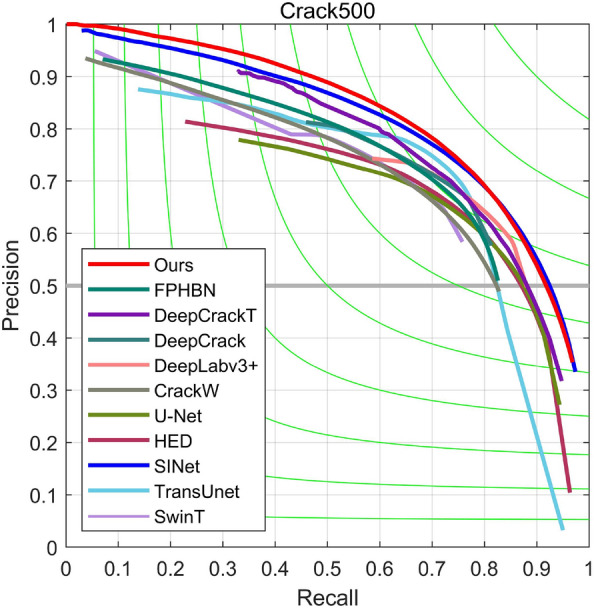
Precision–recall curves on the Crack500 test dataset.

**Figure 8:**
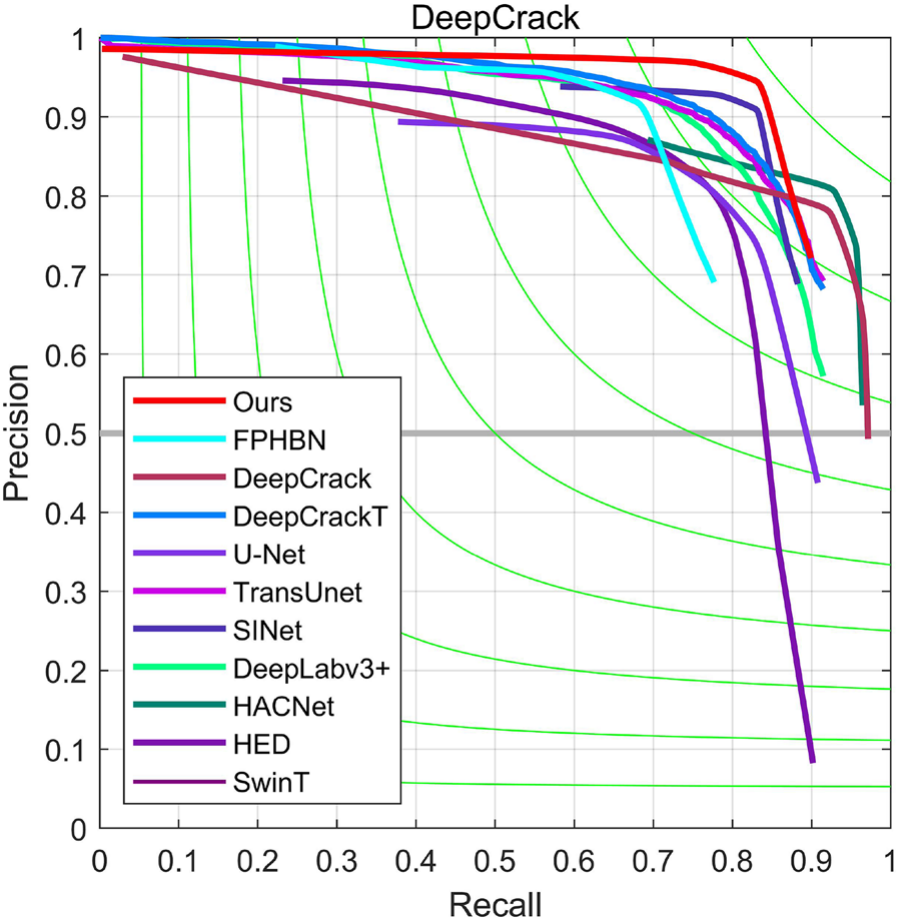
Precision–recall curves on the DeepCrack test dataset.

(3) *Results on CRACK500* Figure 7 demonstrates that our proposed method outperforms other methods as it resides closer to the upper right corner of the P–R curves, indicating superior performance. To validate the practicality of the proposed model, quantitative tests were conducted on the Crack500 dataset. Table 3 presents the performance results on the Crack500 test set, where our proposed method achieves leading levels in several evaluation metrics, including $F_{1}$ and *emph* *MIoU*, with values of 0.754 and 0.802, respectively.The HACNet model exhibits a high recall on the CRACK500 dataset, which can be attributed to its architecture maintaining the same spatial resolution throughout. Our method also surpasses the latest TCDNet in terms of accuracy with comparable speed, improving the $F_{1}$ value by 0.9 and *emph* *MIoU* value by 1. When compared with other classical crack detection networks such as DeepCrack, FPHBN, and ECDFFNet, our proposed method exhibits substantially enhanced detection accuracy. Furthermore, the performance of SwinT and TransUnet, based on Transformer network structures, gradually improves with the increase in data volume in the Crack500 dataset. Therefore, the conducted experiments demonstrate that our proposed method can effectively detect crack defects.

(4) *Results on DeepCrack* As demonstrated in Fig. 8, our proposed method outperforms the compared methods, as it resides closer to the upper right corner of the PR curves. This finding confirms the superior performance of the approach. To further validate its superiority, quantitative tests were conducted on the DeepCrack dataset. As shown in the results presented in Table 3, our proposed method achieves state-of-the-art results in several evaluation metrics, including $F_{1}$ and *emph* *MIoU*, with values of 0.890 and 0.898, respectively. In comparison with the latest TCDNet, our method achieves accuracy improvements with comparable speed, resulting in $F_{1}$ and *emph* *MIoU* value improvements of 0.7 and 0.7, respectively. Furthermore, our proposed method significantly enhances detection accuracy when compared to other classical crack detection networks, including DeepCrack, FPHBN, and ECDFFNet. These results demonstrate that our proposed method can effectively detect crack defects.

**Table 3:** The evaluation metrics of competing methods on the Crack500 and DeepCrack dataset.

Methods	CRACK500						DEEPCRACK					
	*P*	*R*	$F_{1}$	*MPA*	*MIoU*	*FPS*	*P*	*R*	$F_{1}$	*MPA*	*MIoU*	*FPS*
HED^9^	0.652	0.741	0.694	0.853	0.755	**53**	0.808	0.772	0.790	0.879	0.817	**57**
DeepLabv3+^30^	0.695	0.731	0.712	0.876	0.769	19	0.804	0.812	0.808	0.902	0.832	20
U-Net^10^	0.743	0.643	0.689	0.886	0.774	38	0.791	0.795	0.793	0.887	0.818	39
FPHBN^12^	0.684	0.723	0.706	0.868	0.764	22	0.840	0.805	0.822	0.898	0.841	21
HACNet^32^	0.667	**0.817**	0.735	0.870	0.781	27	0.926	0.809	0.864	0.902	0.874	31
DeepCrack^11^	0.672	0.761	0.714	0.875	0.768	49	0.919	0.783	0.846	0.890	0.860	47
DeepCrackT^31^	0.701	0.736	0.717	0.882	0.771	21	0.902	0.775	0.834	0.900	0.847	23
SwinT^33^	0.667	0.698	0.682	0.848	0.749	40	0.910	0.687	0.783	0.888	0.818	39
TransUnet^34^	0.720	0.733	0.727	0.867	0.777	11	0.843	0.787	0.814	0.892	0.843	12
ECDFFNet^23^	0.711	0.772	0.740	0.876	0.786	17	0.903	**0.843**	0.872	0.915	0.884	19
TCDNet^14^	0.731	0.759	0.745	0.893	0.791	21	0.943	0.827	0.883	0.916	0.892	25
**Ours**	**0.799**	0.713	**0.754**	**0.903**	**0.802**	18	**0.958**	0.832	**0.890**	**0.923**	**0.898**	19

Significant values are in bold.

## Conclusion

In this paper, an innovative approach for detecting cracks in tunnel linings is proposed. An enhanced attention module that more effectively captures long-range, aggregation-dependent information is initially introduced. This enhancement enables the model to distinguish between characteristics of cracks and lining seams with greater accuracy. Additionally, mixed strip convolution is integrated into the decoding stage to improve the model’s capacity to capture distant contextual information in four directions: horizontal, vertical, left diagonal, and right diagonal. The effectiveness of the proposed method on the Tunnel200 dataset was assessed, demonstrating its accuracy in detecting cracks in tunnel linings-a critical aspect for ensuring safe tunnel operations. Furthermore, the approach was validated on the Crack500 and DeepCrack pavement crack datasets to highlight its robustness.

In future work, further enhancements to the Transformer structure are planned to better capture contextual information related to the structural characteristics of lining seams, thereby improving the model’s performance. Additionally, the development of a multimodal task system that integrates information from various modalities, including video, image, and language, aims to enhance the early detection of tunnel surface defects.

## Data Availability

The dataset that support the findings of this study are openly available at https://github.com/Qiang-Z/Tunnel200-Dataset and https://github.com/yhlleo/DeepCrack/tree/master/dataset. The authors confirm that the data supporting the findings of this study are available are available from the corresponding author, [LangLang], upon reasonable request, and the data also can get from reference materials.
